# Apathy, cognitive function and motor function in Alzheimer's disease

**DOI:** 10.1590/S1980-57642012DN06040007

**Published:** 2012

**Authors:** Salma S. Soleman Hernandez, Thays Martins Vital, Marcelo Garuffi, Angélica Miki Stein, Camila Vieira Ligo Teixeira, José Luiz Riani Costa, Florindo Stella

**Affiliations:** 1 Institute of Biosciences, Universidade Estadual Paulista (UNESP), Department of Physical Education, Physical Activity and Aging Lab (LAFE), Rio Claro SP, Brazil.; 2 Clinic of Geriatric Psychiatry, Universidade Estadual de Campinas (UNICAMP), Campinas SP, Brazil.

**Keywords:** Alzheimer's disease, apathy, cognition, motor activity

## Abstract

The aims of this study were to characterize the presence of apathy in patients with AD, determine the relationship between apathy, motor function and cognitive function, and to verify differences among patients stratified by level of apathy in relation to cognitive and motor abilities.

**Methods:**

A cross-sectional study was conducted of 37 patients with AD. The following tests were used: MoCA, the Frontal Assessment Battery, Verbal Fluency, Clock Drawing Test, Andreotti & Okuma Battery Tests, Sit and Reach, Resistance of Upper Limbs - AAHPERD Battery Test, Sit and Lift Chair and the Apathy domain of the Neuropsychiatric Inventory. After verifying the normality of the data distribution, comparisons were made using Student's *t*-test and the *U* Mann Whitney test; relationships were also assessed using *Pearson*'s and *Spearman*'s correlation coefficients. All analyses were considered to be statistically significant at a p-value of 0.05.

**Results:**

46% of participants in this study showed mild symptoms of apathy. Significant and weak associations were found (p=0.04) between apathy and the attention domain on the MoCA and between apathy and the Walk Test. Analysis of differences in cognitive and motor functions according to participants' level of apathy revealed no significant differences for any of the variables.

**Conclusion:**

Apathy was reflected in attention and the Walk Test, suggesting these variables may be related to cognitive and functional decline in AD patients.

## Abstract (pt)

Os objetivos deste estudo compreendem: caracterizar a presença de apatia em pacientes com doença de Alzheimer (DA); verificar se há relação entre apatia, funções cognitivas e funcionalidade motora dos mesmos e analisar se há diferenças entre os pacientes, separados por nível de apatia, em relação às suas funções cognitivas e funcionalidade motora.

**Métodos:**

Trata-se de um estudo com delineamento transversal. Ao todo 37 pacientes com DA compuseram a amostra. Foram utilizados os seguintes testes: Mini-Exame de Estado Mental, Montreal Cognitive Assessment (MoCA), Bateria de Avaliação Frontal, Teste de Fluência Verbal Semântica, Teste do Desenho do Relógio, Bateria de Testes de Andreotti & Okuma, Teste de Sentar e Alcançar, Resistência de Membros Superiores da AAHPERD, Teste Sentar-se e Levantar-se da Cadeira e o domínio Apatia do Inventário Neuropsiquiátrico. Após verificar a distribuição dos dados as comparações seguiram através dos testes *t* student e *U* Mann Whitney e as relações, através das correlações de *Pearson* e *Spearman*. Todas as análises admitiram nível de significância de 5%.

**Resultados:**

46% dos participantes deste estudo apresentam sintomatologia para apatia em nível leve. Foram verificadas relações significativas e fracas (p=0,04) entre apatia e o domínio atenção do MoCA e entre apatia e o Teste de Caminhar. Ao verificar a diferença nas funções cognitivas e funcionalidade motora segundo o nível de apatia dos participantes não foram encontradas diferenças significativas para nenhuma das variáveis analisadas.

**Conclusão:**

Apatia relaciona-se com a atenção e o Teste de Caminhar sugerindo que estas variáveis podem estar relacionadas com o declínio cognitivo e funcional dos pacientes com esta patologia.

## INTRODUCTION

Recently, the National Institute on Aging and the Alzheimer's Association have proposed a review of the clinical criteria used to diagnose dementia. Of the changes proposed, the institution has suggested that behavioral changes, which include apathy, loss of motivation and initiative, social withdrawal, lack of empathy and diminished interest in former activities, be included as dementia diagnostic criteria.^1^

In recent years, mounting evidence has emerged concerning the presence of apathy in Alzheimer's disease (AD), the most frequent type of dementia among the elderly.^2-7^ The neuropsychiatric symptom of apathy is often seen in this disease. It is estimated that between 36 and 88% of patients present with apathy, even during the initial course of the disease, and that this symptom tends to worsen with increasing severity of AD.^8-10^

Apathy has been associated with functional decline and loss of autonomy in performing activities of daily living.^2,11,12^ In addition, apathy is associated with an increase in cognitive impairment, executive dysfunction,^2,7^ and cortical atrophy.^13^ Such declines may result in a poorer quality of life as well as a higher risk of institutionalization and early death besides an increase in comorbidities among these patients.^14^

In a recent systematic review, Robert et al.^15^ proposed clinical criteria for the diagnosis of apathy in patients with AD. These criteria define the disturbance of apathy as a motivational disorder that involves three levels of responses related to environmental stimuli and the patient. The criteria are as follows: emotional, behavioral and cognitive, which may result in a decline in the affective, cognitive and functional independence of the patient. These authors also reported the need for more specific instruments and a larger number of studies involving this population.

Cholinesterase inhibitors are the most commonly used pharmacological treatment for the disorder and have been shown to have positive effects on the disease process.^16^ However, the cited authors concluded that these studies are limited with respect to the effectiveness of cholinesterase inhibitors in treating apathy. Given this assertion, other non-pharmacological approaches may constitute an important tool for the treatment of this disturbance.

Of note, few studies have investigated patients living in the community where this may be due to the difficulty of studying these populations.

Thus, investigations with patients in the community may be advantageous because the findings from such studies could help patients find additional support to delay the worsening of the disorder. Furthermore, managing apathy may reduce the risk of early institutionalization. Such investigations may also reveal data that aids towards improving or maintaining the autonomy and independence of these patients, resulting in enhanced quality of life.

The objectives of this study included the following:

[a] to describe the presence of apathy in patients with AD living in the community of Rio Claro-SP/Brazil;
[b] to determine whether there is a relationship between apathy and cognitive and motor function in these patients; and
[c] to assess whether there are differences in cognitive and motor function according to level of apathy in patients.

## METHODS

**Participants.** The study participants were part of the Cognitive and Functional Kinesiotherapy Program in Elderly with Alzheimer's disease (Programa de Cinesioterapia Funcional e Cognitiva em Idosos com doença de Alzheimer PRO-CDA), a project that has served the community since 2006 run by the Department of Physical Education of the Biosciences Institute of the Universidade Estadual Paulista at the campus Rio Claro campus-SP, Brazil.

Patients entered the program on the basis of medical referral or by self or caregiver referral in response to print and broadcast media advertisements of the project.

Inclusion criteria were as follows: clinical diagnosis of AD, according to DSM-IV criteria,^17^ confirmed by medical specialists in the region; mild to moderate AD; maintenance of drug treatments prescribed by doctors and adequate motor ability to perform the tests.

A total of 60 patients were invited to participate in the study, 23 of whom were ineligible for not satisfying the criteria established for inclusion or because they were unable and/or unwilling to attend the evaluations.

More specifically, among the 23 patients excluded, 10 were at the severe stage of disease, 3 patients had some impairment that impeded the conducting of the tests, 3 patients refused to participate in the study, 3 patients had no clinical diagnosis of AD, 3 patients presented with a medical problem that was marked at the time the assessment, and 1 patient was not reached.

Therefore, a final total of 37 patients, 24 (68.87%) with mild and 13 moderate (31.13%) AD, according to the Clinical Dementia Rating Scale, were included in the sample.^18,19^ The patients' guardians signed a consent form, which was approved, along with the research project, by the Research Ethics Committee of the Universidade Estadual Paulista, under protocol number 4826.

**Assessment procedure and instruments.** Initially, all patients underwent an evaluation with a medical psychiatrist who had specialized training in Geriatric Psychiatry; this clinician confirmed the clinical diagnosis of Alzheimer's disease. The psychiatrist then conducted a structured interview to obtain socio-demographic data, age, gender, education, disease duration, medication use and general comorbidities.

The second evaluation conducted by a blinded examiner was scheduled. The patient was evaluated for cognitive and motor functioning, described in more detail below. All evaluations were performed in the afternoon within a quiet environment.

Global cognitive assessment included the following tests:

- The Mini-Mental State Examination (MMSE) is an instrument consisting of questions grouped into seven categories that include the following areas: orientation, immediate memory, attention and calculation, delayed recall and language. The MMSE score ranges from 0 to 30 points.^20,21^
- The Montreal Cognitive Assessment (MoCA) is an instrument developed to screen for mild cognitive deficits. This test assesses different cognitive domains, including executive visuospatial memory, naming, attention, language, abstraction, delayed recall and orientation. The total score is 30 points.^22,23^
- The Frontal Assessment Battery (FAB) is a scale developed to assess frontal cognitive functions and it was recently proposed as a brief tool for determining diagnosis in cases of executive dysfunction. The battery consists of six subtests, which are as follows: similarities (abstract reasoning), lexical fluency, motor series (programming for cognitive motor action), conflicting instructions (sensitivity to interference), inhibitory control and handgrip strength.^24,25^
- The Semantic Verbal Fluency (VF) test measures the ability of the subject to name as many animals as possible within one minute. This test primarily measures processing speed, semantic memory and language.^26^
- The Clock Drawing Test (CDT) is designed to assess executive functions (i.e., planning, abstract thought, logic and monitoring of executive processing).^27^

The apathy domain of the Neuropsychiatric Inventory (NPI-apathy) was used to measure apathy. This instrument was developed to assess psychopathology in patients with dementia. It also identifies the frequency and intensity of Apathy, among others, based on questions administered to the patient's caregiver. The score is determined by multiplying the frequency by the intensity of behaviors assessed and lies within a range of between 1 and 12 points. Moreover, this instrument evaluates the burden of caregiver-related disorders assessed in the patient.^28^

The assessment of motor function was conducted using the Andreotti & Okuma battery of tests.^29^ This battery consists of subtests that assess aerobic capacity, agility, and hand-eye coordination, as well as several specific activities of daily living in the elderly.

- Walk 800 meters (WAL).
- Sit and get up from a chair and move around the House (MH).
- Manual Abilities (MA).
- Climbing Stairs (CS).
- Rising from the Ground (RG).
- Putting on Socks (PS).
  For a complete evaluation of functionality, the following tests were included:
- Sit and reach (SR), to measure the flexibility of patients.^30^
- Resistance of the Upper Limbs (RUL) from the AAHPERD battery of tests.^31^
- Sit and Get up from a chair in 30 seconds (SGC) was used to evaluate the resistance of the lower limbs and was conducted based on the steps set forth by Rikli & Jones.^32^ In the test, male and female patients held a medicine ball weighing 3 kg and 2 kg, respectively.

**Statistical analysis.** The *Shapiro Wilk* test was used to verify the data distribution. For normally distributed data, *Pearson*'s correlation index was applied, whereas *Spearman*'s correlation index was employed for data that were not normally distributed.

The data that were normally distributed included the following: orientation and total score on the MMSE; executive visuospatial memory, attention and total score on the MoCA; the FAB; the VF; the SR and the NPI-Apathy. The data that were not normally distributed were as follows: immediate memory, attention and calculation, recall and language on the MMSE; naming, language, abstraction, delayed recall and orientation on the MoCA; similarities, lexical fluency, motor series, conflicting instructions, inhibitory control and handgrip strength for the FAB, CDT, WAL, MH, CS, RG, MA, PS, RUL and RML.

In a secondary analysis, patients were divided into subgroups. Because of the small sample size, subgroups were determined based on the mean score obtained on the NPI-apathy. The groups were as follows: those scoring greater than or equal to the mean were assigned to the apathetic group (AG), and those who scored below the mean were placed in the non-apathetic group (NAG). The groups were subsequently compared using Student's t test for normally distributed data and the *U* Mann Whitney test for non-normally distributed data. Descriptive statistics were also used to illustrate the data analyzed. All analyses were considered to be statistically significant at a p-value of 0.05.

## RESULTS

Of the 37 patients who participated in the study, 29 (78.38%) were female and 8 (21.62%) male, 24 (68.87%) were at a mild AD stage and 13 (31.13%) moderate. Subjects had a mean age of 78.8±7 ye, 4.4±3.6 years in education, 17.2±4.4points on the Mini Mental State Examination, 29.8±30.5month disease duration and 2.7±3.^1^ points on the apathy domain of the Neuropsychiatric Inventory.

Table 1 shows baseline clinical and socio-demographic characteristics of the sample.

**Table 1:** Clinical and socio-demographic characteristics of study participants with AD (n=37). Means and standard deviations of the following variables: age, education, gender, stage of disease, Mini-Mental State Examination, disease duration and average score on the Neuropsychiatric Inventory.

Characteristics of patients with AD (n=37)	
Age (years)	78.8±7
Education (years)	4.4±3.6
Female	29 (78.38%)
Male	8 (21.62%)
CDR 1	24 (68.87%)
CDR 2	13 (31.13%)
MMSE (points)	17.2±4.4
Disease duration (months)	29.8±30.5
NPI-apathy (points)	2.7±3.1

CDR: Clinical Dementia Rating; MMSE: Mini Mental State Examination; NPI-apathy: apathy domain of the Neuropsychiatric Inventory.

For the application of the motor battery, some adjustments were made, such as modifications of the instructions for the tests.

Despite adjustments, of the 37 patients, 4 failed to complete or perform the walking test, three were unable to move around the house (agility), two were unable to rise from the ground, two could not put on socks, four did not complete the flexibility test, two did not perform the test for strength of the lower limbs, and one did not complete the upper limbs tests. These subjects were not included in the statistical analysis.

Correlation analysis showed a significant relationship between apathy and attention (MoCA domain) and between apathy and Test Walk 800 meters.

The relationships found between apathy (Apathy-NPI) and the MMSE test; MoCA; FAB; VF; CDT, WAL, MH, MA, CS, RG, PS, SR, RUL, SGC are given in the table below (Table 2).

**Table 2:** Relationship between apathy, as measured by NPI-apathy, and MMSE tests, MoCA, VF, CDT, WAL, MH, MA, CS, RG, PS, SR, RUL, and SGC for 37 study participants.

**Mini-Mental State Examination - MMSE**									
		MMSE total score	Orientation	Immediate memory	Attention/ calculation	Delayed recall	Language		
	NPI-Apathy	0.1	0.1	-0.0	0.1	0.0	0.1		
**Montreal Cognitive Assessment - MoCA**									
		MoCA total score	Visuospatial memory exec.	Naming	Attention	Language	Abstraction	Delayed recall	Orientation
	NPI-Apathy	0.2	0.1	0.23	-0.3*	0.2	-0.1	-0.0	0.2
**Frontal Assessment Battery - FAB, Verbal Fluency - VF and Clock Drawing Test - CDT**									
	FAB total score	Similarities	Lexical fluency	Motor series	Conflicting instructions	Inhibitory control	Handgrip strength	VF	CDT
NPI-Apathy	0.2	-0.0	0.0	0.3	0.0	0.1	0.1	0.1	0.2
**Battery of Tests to Assess Motor Function**									
	Walking	Move around house	Manual abilities	Climbing stairs	Rising from ground	Putting on socks	Sit and reach	Resistance upper limbs	Sit and get up from chair
NPI-Apathy	-0.3*	-0.2	-0.2	-0.2	-0.3	-0.3	-0.1	0.1	0.0

* p<0.05

The mean score obtained on the NPI-apathy scale by participants in the study was 2.7 points. Of the overall sample, 17 cases (46%) had a score higher than the mean, forming the non-apathetic group (NAG), and 20 (54%) had a score lower than the mean, forming the apathetic group (AG).

Comparison of the groups for cognitive ability and motor function based on patients' level of apathy found no statistically significant differences for any of the variables.

Table 3 shows the comparison between the two apathy groups. Statistical analyses detected no significant differences for any of the variables between the groups.

**Table 3:** Mean scores and standard deviations on tests administered for apathetic (AG, n=17) and non-apathetic (NAG, n=20) groups: MMSE, MoCA, FAB, VF, CDT, WAL, MH, MA, CS, RG, PS, SR, RUL, SGC and NPI-apathy.

Tests	AG	NAG
MMSE (total score)	17.8±5.2	16.6±3.9
Orientation (n/10)	5.5±2.8	4.9±1.7
Immediate Memory (n/3)	2.7±0.8	2.9±0.4
Attention and Calculation (n/5)	2.2±1.7	1.6±1.5
Delayed Recall (n/3)	0.4±1.0	0.2±0.5
Language (n/9)	7.0±1.3	6.8±1.3
MoCA (total score)	12.3±5.6	10.2±4.8
Executive Visuospatial Memory (n/5)	2.1±1.5	1.8±1.0
Naming (n/3)	1.7±0.9	1.7±0.8
Attention (n/6)	3.3±1.4	2.3±1.8
Language (n/3)	1.1±1.2	0.8±0.7
Abstraction (n/2)	0.2±0.4	0.6±0.7
Delayed Recall (n/5)	0.2±0.4	0.5±0.8
Orientation (n/6)	3.4±1.8	2.3±1.3
Frontal Assessment Battery (total score)	11.5±4.1	10.2±4.2
Similarities (n/3)	1.8±0.8	1.7±1.0
Lexical Fluency (n/3)	1.4±1.1	1.5±1.0
Motor Series (n/3)	2.2±1.1	1.7±1.3
Conflicting Instructions (n/3)	1.8±1.1	1.7±1.3
Inhibitory Control (n/3)	1.4±1.2	1.0±1.0
Handgrip Strength (n/3)	2.7±0.7	2.5±0.9
Verbal Fluency	6.9±3.5	6.3±3.7
Clock Drawing Test (n/10)	5.5±3.1	4.6±2.1
Walk 800 meters (s)	692.6±221.2	858.9±351.8
Sit and get up from chair and move around House (s)	71.3±37.6	73.3±35.3
Manual Abilities (s)	23.1±16.5	26.9±12.7
Climbing Stairs (s)	15.9±7.6	22.1±15.9
Rising from Ground (s)	24.5±25.8	22. 7±24.8
Putting on Socks (s)	11.6±10.8	10.5±8.3
Sit and reach (cm)	15.4±7.6	18.3±10.9
Resistance Upper Limbs (repetitions)	15.5±4.9	15.2±3.4
Test Sit and Get up from chair (repetitions)	10.5±2.1	10.9±3.8
NPI-Apathy	5.4±2.6	0.3±0.5

## DISCUSSION

**Apathy profile and assessment.** According to the average score measured by the NPI - Apathy (17), 46% of the subjects in this study had symptoms of apathy. These findings corroborate the results of the study by Tatsch et al.^32^ which found a prevalence of 56.1% in 41 patients with AD living in a community of São Paulo.

Another important factor to note is that the scales used to identify the presence of apathy were primarily answered by the caregiver, who is often overloaded and stressed. Thus, caregivers may not be able to distinguish the symptoms listed above and are potentially more likely to overestimate symptoms in their family members 34.

By contrast, Rockwood^35^ advocates a broad approach using research with regard to the evaluation of individuals with dementia. According to this author, patients and caregivers have information that is as relevant for the scales and can be used for evaluation.

Given the circumstances described above, it seems that the diagnosis of apathy requires an understanding of the relationship between observation, patient response, the response of the caregiver and a clinical evaluation obtained by specialists.

To this end, a scale widely used in a clinical setting, the Neuropsychiatric Inventory (NPI), was restructured by Medeiros and colleagues^36^ to obtain this understanding for a broad diagnosis. The *Neuropsychiatric Inventory-Clinician* (NPI-C) *rating scale* includes the clinical version for the diagnosis of behavior (beyond the response of the caregiver and patient) and can be considered for the diagnosis of apathy in patients with AD.

**Apathy and cognitive function.** As previously outlined, apathy may be related to decreased cognitive functioning and/or increased functional decline, loss of autonomy and the ability to perform activities of daily living in this patient population. In this study, a weak relationship was found between apathy and the attention domain of the MoCA.

Given this relationship, impaired attention may be an indicator of the presence of apathy in community-dwelling patients.

According to Perry & Hodges,^38^ attentional deficits can be considered the first cognitive domain, unrelated to memory, to decline in AD. In addition, apathy may be present even in early stages of the disease.^9^

Given the variety of scales applied and the number of subdomains comprising these scales, we expected to find a greater number of relationships, especially with variables that assess frontal cognitive functions, as apathy has been related to dysfunction of the circuitry involving the prefrontal cortex.^4,8,39^

This finding may be due to the relatively small sample size used and the relative preservation of cognitive functioning, functional independence and behavioral symptoms in the participating patients.

**Apathy and motor function. **Some data suggest that apathy may explain, at least in part, the functional decline in AD patients, which results in worsening of the ability to perform activities of daily living.^11,12^

Consistent with these data, we found a weak relationship between apathy and the 800-meter test walk. The practice of walking moderate distances is considered one of the most frequent activities among the elderly.^29^ This relationship may represent a landmark in the decline of motor function among elderly people with AD.

When analyzing the patients, divided by level of apathy, the statistical analyses indicated no significant differences between groups for cognitive and motor functioning. Again, this could be related to the low number of participants and the relative preservation of cognitive functioning, functional independence and behavioral symptoms in the patients studied.

These results seem to indicate that although patients living in the community have symptoms of apathy, they exhibit this symptom in a more subdued manner in comparison with those patients already institutionalized. This reinforces the need for more research on the disorder in this population.

In a more comprehensive analysis, scores obtained by the apathetic (AG) and non-apathetic (NAG) groups for the MMSE and MoCA were examined. It can be observed that scores by both groups were more impaired for delayed recall, abstraction and visuospatial memory in relation to other executive subdomains measured. These results seem to indicate a decline in most frontal areas and may be related to the disturbance of apathy in AD, as it involves the frontal brain circuitry.^4,8,38^

With respect to the scores obtained by the groups for the FAB, lexical fluency and inhibitory control yielded the lowest scores. These data indicate that mental flexibility and autonomy in internal control of environmental stimuli were more damaged when compared to other domains assessed by this scale. In addition, although relationships were not identified for this study, these variables may be related to the disturbance of apathy in patients with AD.^15^

The motor function data for patients in this study highlight the difficulty of assessing functionality in AD patients. A total of 60 patients were invited to participate in this study, 23 of whom were ineligible to participate for not meeting the criteria established for inclusion in the study or because they were unable and/or unwilling to attend the evaluations.

Although the exclusion of these patients obeys the inclusion criteria, we reaffirm this as a limitation of the study. Did patients unable or unwilling to perform the proposed motor tasks include those with greater apathy? Thus, alerted to this fact, future studies should be able to overcome this limitation.

The battery of tests proposed for this study encompasses the main daily activities performed by older adults.^29^

Little is known about the profile of motor function in AD patients. It is generally believed that it declines with advancing disease^40,41^ but there is relatively scant data concerning comparisons of motor function and/or the establishment of standards. The data reported, while not standard for motor function in AD patients, offer more in as far as they aggregate the experience of practitioners and the clinical observations of these patients by the authors.

There was a wide variation in the time taken to perform the activities proposed by the battery. The stand to walking test, which took between 576 to 1447 seconds to execute (Δ=871), strengthens the relationship found between apathy and the test in question.

This relationship appears to indicate that loss of autonomy and independence-related apathy may be related to gait or lower body strength of the AD patients in this study. Given the relative functional preservation of our sample, could walk or gait in this population be considered an indicator of functional symptomatology of apathy in AD?

Similarly, the tests assessing the patients' ability to move around the house and rise from the ground, as well as manual abilities, showed wide variations in execution time: 37.4 to 166 (Δ=128.6), 3.6 to 108 (Δ=104. 4) and 8.6 to 76 (Δ=67.4) seconds, respectively. Other tests did not identify major differences in implementation.

In general, we emphasized the activities of walking, movement around the house, rising from the ground and performing manual tasks, as they are more markedly impaired in patients residing in the community and who have mild to moderate stages of disease.

In summary, the symptoms of apathy in community-dwelling AD patients occur at a mild degree of severity. The evaluation of apathy in AD should be conducted considering a wide range of functions, including cognitive function, behavioral function and activities of daily living. Such evaluations should also include patients, caregivers and experts.

In our sample of community-dwelling AD patients, apathy was associated with attention deficits and a decline in the ability to walk. The decline in other functions, such as moving around the house (agility), rising from the ground (lower limb strength and flexibility) and performing manual activities may also be related to the disturbance of apathy in AD.

Of the 37 patients with AD living in the community of Rio Claro-SP/Brazil, 17 (46%) had symptoms of mild apathy. The apathy observed was reflected in attention and the outcome of the walk test, suggesting that these variables may be related to cognitive and functional decline in patients with this pathology. There were no differences between patients stratified by level of apathy for cognitive and motor functioning, likely due to the relatively high level of preservation of the patients and the small sample used in the study.

The 37 study participants showed preservation of their cognitive, motor and behavioral functioning probably due to disease stage. The average score obtained by patients for Apathy was 2.7 points out of a maximum of 12. Thus, it can be concluded that subjects had mild symptoms, which may explain the few relationships found.

According to Starkstein et al.,^34^ the prevalence of apathy in AD can vary greatly due to the overlap of depression with apathy. In addition, in their study, the lack of structured instruments for diagnosing apathy was a confounder. Could we have possibly excluded/confounded apathetic and depressed patients ?

Moreover, it is possible that no differences were found due to lack of statistical power (with the limited sample size) or to sample selection (only elderly able to perform physical tests were included, perhaps excluding the most apathetic). Further studies should be able to overcome these limitations.
